# Dendronized chitosan hydrogel with GIT1 to accelerate bone defect repair through increasing local neovascular amount

**DOI:** 10.1016/j.bonr.2023.101712

**Published:** 2023-08-30

**Authors:** Lin Cheng, Zhimin Zhou, Qingqing Li, Wen Li, Xin Li, Gen Li, Jin Fan, Lipeng Yu, Guoyong Yin

**Affiliations:** a Department of Orthopedics, The Affiliated Hospital of Xuzhou Medical University, Huaihai West Road 99, Xuzhou, Jiangsu Province 221000, China; b Department of Orthopedics, The First Affiliated Hospital of Nanjing Medical University, Guangzhou Road 300, Nanjing, Jiangsu Province 210000, China; c School of Materials Science and Engineering, Shanghai University, Nanchen Street 333, Shanghai 200444, China

**Keywords:** Injectable hydrogel, Bone regeneration, Mesenchymal stem cells, GIT1, Notch signaling

## Abstract

Bone defects have long been a major healthcare issue because of the difficulties in regenerating bone mass volume and the high cost of treatment. G protein-coupled receptor kinase 2 interacting protein 1 (GIT1) has been proven to play an important role both in vascular development and in bone fracture healing. In this study, a type of thermoresponsive injectable hydrogel from oligoethylene glycol-based dendronized chitosan (**G1-CS**) was loaded with GIT1-plasmids (**G1-CS**/GIT1), and used to fill unicortical bone defects. RT-PCR analysis confirmed that **G1-CS**/GIT1 enhanced DNA transfection in MSCs both *in vitro* and *in vivo*. From the results of micro-CT, RT-PCR and histological analysis, it can be concluded that **G1-CS**/GIT1 accelerated the bone healing rate and increased the amount of neovascularization around the bone defects. In addition, an adeno-associated virus (AAV)-GIT1 was constructed to transfect mesenchymal stem cells. The results of capillary tube formation assay, immunofluorescence staining and western blot analysis proved that high expression of GIT1 induces mesenchymal stem cells to differentiate into endothelial cells. RT-PCR analysis and capillary tube formation assay confirmed that the Notch signaling pathway was activated in the differentiation process. Overall, we developed an efficient strategy through combination of injectable hydrogel and G1T1 for bone tissue engineering.

## Highlights

• G1-CS/GIT1 significantly improve local GIT1 expression by minimally-invasive surgery.
• High expression of GIT1 promotes osteogenesis and accelerates bone defect repair.
• GIT1 induces MSCs to differentiate into ECs, increasing the local vasculature, which contributes to bone defect repair.
• Notch signaling pathway is activated during the differentiation procedure.

## 1 Introduction

The treatment of bone defects resulting from trauma, infection, tumors or congenital deficiency is still a major healthcare issue due to the difficulties in regenerating mass bone volume (Feng et al., 2021; Baker et al., 2016). The expense of treatment is a significant burden for patients and the outcomes are not very satisfactory. Methods used to repair bone defects usually include autografts, allografts, cell-seeded allograft implants and synthetic bone graft substitutes. Autografting is the best way to treat bone defects, but the available bone volume is limited (Coyac et al., 2021; O'Keefe and Mao, 2011). Allografts can provide mass bone volume, but immune rejection often causes resorption of the transplanted bone and results in failure of osseointegration with a rate of 60 % at 10 years (Chiarello et al., 2013; Xie et al., 2007). Synthetic bone graft substitutes including calcium phosphate, calcium sulfate fillers and cements have been developed, but these normally have poor mechanical strength and fracture resistance compared to autografts or allografts (Baldwin et al., 2019; Babiker, 2013; Zhang et al., 2008).

In recent years, hydrogels with water-swollen, biocompatible and 3D polymer network structures have been considered as very promising biomaterials for tissue engineering. In particular, injectable hydrogels have been widely used for delivery of cells, drugs, and other bioactive substances, due to their attractive features including homogeneous distribution of cells and molecules regardless of the defect size and shape, and the minimally-invasive delivery procedure (Wang et al., 2017; Tseng et al., 2015). They have been demonstrated to be suitable scaffolds for neural tissue engineering (He et al., 2019; Li et al., 2018), stem cell delivery (Han et al., 2019; Koivusalo et al., 2019), and bone regeneration (Deng et al., 2020; Wang et al., 2019; Luo et al., 2019). There was also a study which demonstrated that a hydrogel could significantly enhance gene transfection efficiency (Lin et al., 2017). By incorporating both mesenchymal stem cells (MSCs) and transfection agents into hydrogels, it is possible to successfully transfect encapsulated MSCs *in situ (*Carthew et al., *2020**)*. However, most of these hydrogels have been used only as carriers of bioactive substances, with no benefit for tissue regeneration by themselves.

G protein-coupled receptor kinase 2 interacting protein 1 (GIT1) is a multi-domain scaffold protein expressed in many cell types including neurons, endothelial and vascular smooth muscle cells, osteocytes, osteoblasts and osteoclasts. The major functions of GIT1 involve actin cytoskeleton organization, receptor trafficking and endocytosis (Menon et al., 2010). It has been proved that GIT1 is a key mediator of bone homeostasis. Macrophage GIT1 contributes to bone regeneration by regulating inflammatory responses (Zhao et al., 2020). Platelet-derived growth factor (PDGF) can rapidly stimulate GIT1 expression in osteoblasts, increase osteoblast proliferation and inhibit cell apoptosis (Wu et al., 2014). GIT1 deficiency leads to decreased revascularization of the fracture callus, decreased chondrocyte proliferation and apoptosis, and reduced osteoclast number (Yin et al., 2014; Chen et al., 2017). As GIT1 has an established role in bone fracture healing, we assumed that high expression of GIT1 would promote bone defect healing.

**Fig. 1:**
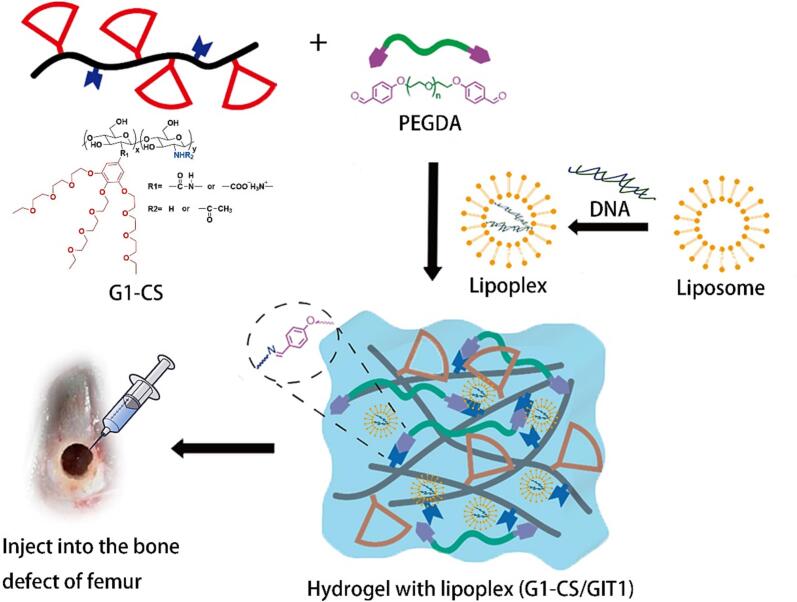
Schematic representation of the preparation of **G1-CS** hydrogel and lipoplex complex (**G1-CS**/GIT1), and injection into the bone defect in a rat femur.

We previously engineered one type of dendronized chitosan (**G1-CS**) by modifying chitosan with an OEG-based dendron (Zhang et al., 2019a). **G1-CS** is water soluble at room temperature, and can form an injectable hydrogel with shear thinning property through Schiff-base chemistry after addition of a crosslinker (**PEGDA**) (Fig. 1). In particular, after injection under physiological conditions, a hydrogel with significantly enhanced mechanical properties was obtained due to the enhanced hydrophobic interactions caused by the thermo- and salt-responsive properties of **G1-CS**. We demonstrated that MSCs encapsulated in the **G1-CS** hydrogel maintained their potential to differentiate towards the osteogenic lineage, which was beneficial for bone defect healing. In the present work, as shown in Fig. 1, we constructed a unicortical bone defect model, and plasmid-GIT1 carried by the **G1-CS** hydrogel (**G1-CS**/G1T1) was applied as an injectable scaffold to fill the bone defects. The effects of this complex system on bone defect healing were thoroughly evaluated by micro-CT, RT-PCR analysis and histological analysis. Furthermore, the mechanism of action of **G1-CS**/G1T1complex on promoting healing of bone defects was studied through capillary tube formation assay, immunofluorescence staining, western blot analysis and RT-PCR analysis.

## 2 Results and discussion

### 2.1 *In vitro* and *in vivo* DNA transfection

**Fig. 2:**
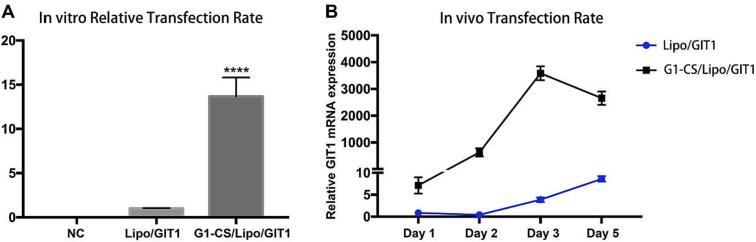
Gene transfection rate of MSCs transfected with or without **G1-CS** hydrogel. **A)***In vitro* transfection rate, Lipo/GIT1 was regarded as the control group. **B)***In vivo* transfection rate, all gene expression was compared to that of the Lipo/GIT1 group at Day 1. ^⁎⁎⁎⁎^*p* < 0.0001.

DNA transfection of stem cells has always been a great challenge. Their low genetic transfection efficiency limits the application of cationic liposomes. Electroporation has high transfection efficiency (Kim et al., 2019; Kumar et al., 2019; Potter and Heller, 2017; Bugeon et al., 2017), but has the disadvantage of also causing high cell mortality (Zhang et al., 2018). Recently, researchers found that hydrogel could enhance the DNA transfection rate when cells and DNA were co-encapsulated (Pannier et al., 1943). In our previous work, we proved that dendritic OEG-modified chitosan (**G1-CS**) aqueous solution could be mixed with stem cells at room temperature, and form a hydrogel with shear thinning property after addition of crosslinkers. When injected into culture medium under physiological conditions, a hydrogel with high stability and improved mechanical properties was generated which supported cell proliferation and differentiation. Here, the DNA transfection into stem cells by **G1-CS** hydrogel was first investigated. We constructed plasmids loaded with GIT1 DNA and its negative control (NC). We transfected 293 T cells with plasmid-GIT1, and extracted total protein from the cells at 48 h post-transfection. Protein expression was then analyzed by western blotting. As shown in **Fig. S1**, GIT1 expression of transfected cells was much higher than that of vehicle control (VC) or NC groups. Then we used the plasmid-GIT1 to transfect mesenchymal stem cells (MSCs) with or without **G1-CS** hydrogel, and extracted the total RNA from cells or cell-hydrogel mixtures at 48 h post-transfection. GIT1 gene expression of MSCs transfected with **G1-CS** hydrogel increased more than ten-fold over that of MSCs transfected without **G1-CS** hydrogel, as shown in Fig. 2A. We also repeated this procedure *in vivo*, and found that GIT1 gene expression of the bone defect filled with **G1-CS** hydrogel and plasmids increased significantly at the second day postsurgery, and was >3000 times higher at day 3 compared to that of Lipo/GIT1 at day 1. Meanwhile GIT1 gene expression was increased only 8-fold at 5 days after transfection in the Lipo/GIT1 group, as shown in Fig. 2B. There was a significant difference between the two groups (*p* < 0.05). These results suggested that **G1-CS** hydrogel enhanced DNA transfer both *in vitro* and *in vivo*, which is consistent with other studies (Carthew et al., 2020; Paidikondala et al., 2019; Ma et al., 2015). We hypothesized that there are two reasons for the enhancement of DNA transfection with **G1-CS** hydrogel. First, the **G1-CS** hydrogel contained positively-charged amine groups, which could increase the concentration of DNA around cells and simultaneously improve the function of transfected DNA. In addition, as a soft material with relatively low elastic modulus, **G1-CS** hydrogel could reduce the membrane shear modulus of cells cultured on or in it, allowing the cell membrane to become easier to deform (Major et al., 2019; Guo et al., 2017), thus enhancing DNA endocytosis.

### 2.2 *In vivo* bone defect healing

**Fig. 3:**
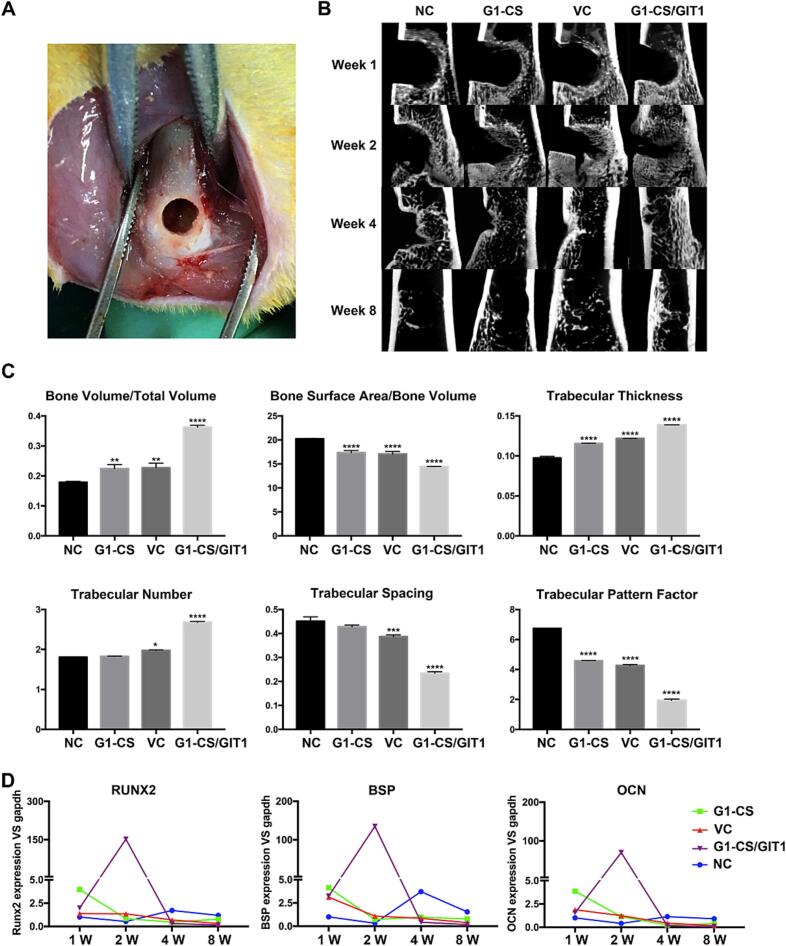
A) Bone defect model in the rat distal femur. B) Micro-CT images of the defects at different time-points in the NC, ****G1-CS****, VC and ****G1-CS****/GIT1 groups. C**)** Quantitative micro-CT analysis of the NC, ****G1-CS****, VC and ****G1-CS****/GIT1 groups at week 4. D**)** Osteogenic marker expression in the NC, ****G1-CS****, VC and ****G1-CS****/GIT1 groups at different time-points. ^⁎⁎^*p* < 0.01; ^⁎⁎⁎^*p* < 0.001; ^⁎⁎⁎⁎^*p* < 0.0001.

To evaluate the *in vivo* effect of **G1-CS**/GIT1 on bone regeneration, we used a well-established bone defect model (Fig. 3A) (Li et al., 2009). Rats were randomly divided into four groups. The bone defects were untreated in the NC group, filled with **G1-CS** hydrogel in the **G1-CS** group, packed with **G1-CS** hydrogel and empty plasmids in the vehicle control (VC) group, and loaded with **G1-CS** hydrogel and plasmid-GIT1 in the **G1-CS**/GIT1 group. At 1, 2, 4 and 8-weeks postsurgery, the defects were scanned by micro-CT (Siemens AG, Erlangen, Germany) at a resolution of 9.5 μm and the results are shown in Fig. 3B. At week 1, no new bone was detected in any of the four groups. At week 2, the **G1-CS** group, VC group and **G1-CS**/GIT1 groups all showed bone ingrowth near the bottom of the defect zone, especially the **G1-CS**/GIT1 group, while in the NC group no new tissue formation was evident in the defect zone. At week 4, substantial new bone formation was observed in all of the four groups, with the most extensive new bone formation observed in the **G1-CS**/GIT1 group. The bone volume of the NC group was still less than that of the other three groups. However, by week 8, the bone defects in all four groups had healed.

Quantitative micro-CT analysis at week 4 (Fig. 3C) showed that the volume of new bone formation per total hydrogel volume (BV/TV, %), the trabecular thickness (Tb. Th), and the trabecular number (Tb. N) in the **G1-CS** group, VC group and **G1-CS**/GIT1 group were all significantly higher than that of the NC group (*p* < 0.01), and the **G1-CS**/GIT1 group exhibited the highest value. The bone surface area/bone volume (BSA/BV), trabecular spacing (Tb. S), and trabecular pattern factor (TPF) were all notably lower in the **G1-CS** group, VC group and **G1-CS**/GIT1 group than in the NC group (*p* < 0.01).

The mRNA expression of both an early stage osteogenic marker (runt related transcription factor 2 (Runx2)) and late stage mineralization markers (osteocalcin (OCN) and bone sialoprotein (BSP)) were monitored at four time points during the 8-week implantation. As shown in Fig. 3D, the mRNA expression levels of Runx2, BSP and OCN were enhanced at week 2 in the **G1-CS**/GIT1 group, and at week 4 in the NC group, and the expression peak of the NC group was much lower than that of the GIT1 group. In the **G1-CS** and VC groups, the expression of the three key osteogenic markers were higher than those of the NC group up to 2 weeks, but became lower after 4 weeks. These results indicated that GIT1 upregulated multiple osteogenic factors at various stages of osteoblastic differentiation to enhance bone formation.

**Fig. 4:**
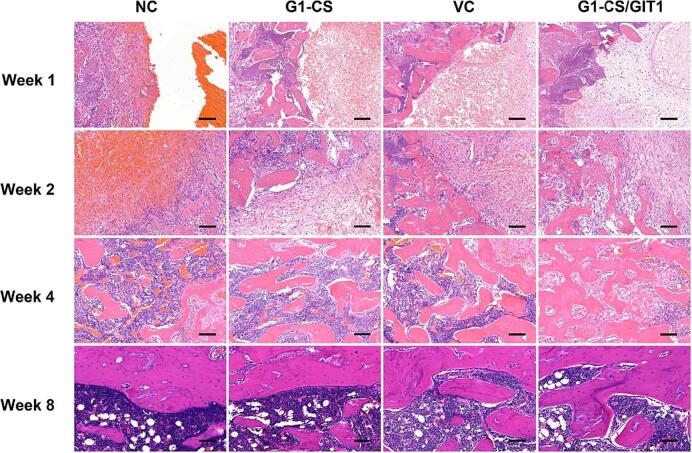
Hematoxylin and eosin staining of the defect area of the NC, ****G1-CS****, VC and ****G1-CS****/GIT1 groups at different time points. The scale bar represents 100 μm.

After micro-CT analysis, the femurs were decalcified with 10 % EDTA, and subsequently embedded in paraffin. Coronal sections 5 μm thick were prepared, and histological analysis was carried out to further analyze the new bone tissue formation. The results of HE staining are shown in Fig. 4. At week 1, the defects in the NC group were mainly filled with blood cells, while the bone defects in the other three groups were mainly filled with **G1-CS** hydrogel, with blood cells filling the pores of the hydrogel. At week 2, a large number of blood cells and some fibrous cells were still present in the defects of the NC group, while the formation of new cartilage and trabeculae could be seen in the other three groups. The **G1-CS**/GIT1 group had the largest amount of new bone volume. Residual hydrogel could still be seen in all three treated groups. At week 4, some new bone formation was observed in the NC group, while the number of bone trabeculae in all three treated groups increased compared to that at week 2. The **G1-CS**/GIT1 group had the largest number of bone trabeculae, and the hydrogel used to fill the defect had been completely absorbed. By 8 weeks after surgery, all the bone defects in the four groups had healed and mineralized.

**Fig. 5:**
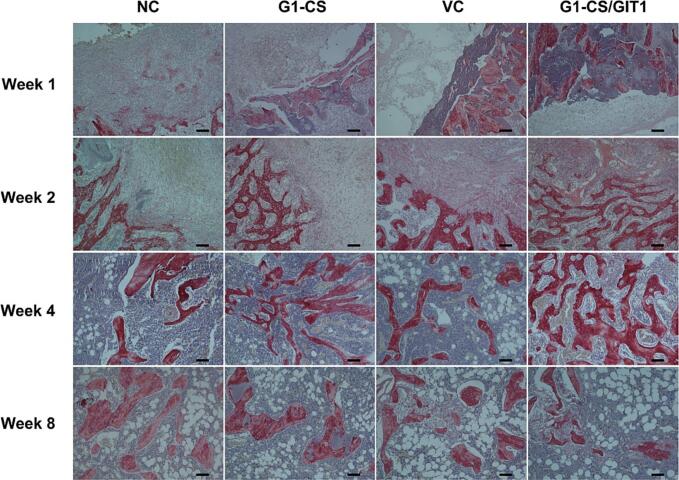
Total collagen staining of the defect area of NC, ****G1-CS****, VC and ****G1-CS****/GIT1 groups at different time-points. The scale bar represents 100 μm.

The results of total collagen staining and ALP staining are shown in Fig. 5 and **Fig. S2**. Total collagen mainly reflected the mineralized collagen in bone tissue, and the formation of new bone was observed. As shown in Fig. 5, there was no significant positive staining in any of the four groups at week 1. At week 2, there was still no positive staining in the NC group, while there was positive staining in the other three groups, and the strongest positive staining was observed in the **G1-CS**/GIT1 group. At week 4, the positive staining in the **G1-CS**, VC and **G1-CS**/GIT1 groups was increased compared to that of week 2. Positive staining was found in the NC group, but less than the other three groups, while the **G1-CS**/GIT1 group still had the strongest positive staining. At week 8, the bone defects of all four groups had healed and the mineralized collagen was distributed evenly. ALP staining reflects osteogenic activity, and the formation of new bone can be observed. As shown in **Fig. S2**, no significant positive staining was found in any of the four groups at week 1. At week 2, no positive staining was found in the NC group, while brown or black positive staining was found in the other three groups, with the strongest positive staining found in the **G1-CS**/GIT1 group. At week 4, the positive staining in the **G1-CS**, VC and **G1-CS**/GIT1 groups were all much more than that of week 2. Positive staining was also observed in the NC group, but less than the other three groups, while the **G1-CS**/GIT1 group still had the strongest staining. At week 8, all the bone defects in all four groups had healed, and the osteogenic activity decreased.

The above results suggested that **G1-CS** hydrogel accelerated the healing of bone defects in this model, but did not affect the final outcomes. This may be due to the network structure of the **G1-CS** hydrogel which is conducive for the proliferation of MSCs (Gibbs et al., 2016), and the biodegradation behavior which allowed the hydrogel to be replaced by newly-formed tissues (Zhang et al., 2019b). In our previous study, we also proved that the **G1-CS** hydrogel-induced MSCs were more likely to differentiate into osteogenic cells, which was beneficial for bone defect healing (Zhang et al., 2019a). We also found that the healing rate of bone defects in the **G1-CS**/GIT1 group was faster than that in the VC and **G1-CS** groups, indicating that GIT1 may play an important role during bone defect healing.

### 2.3 Local neovascularization

**Fig. 6:**
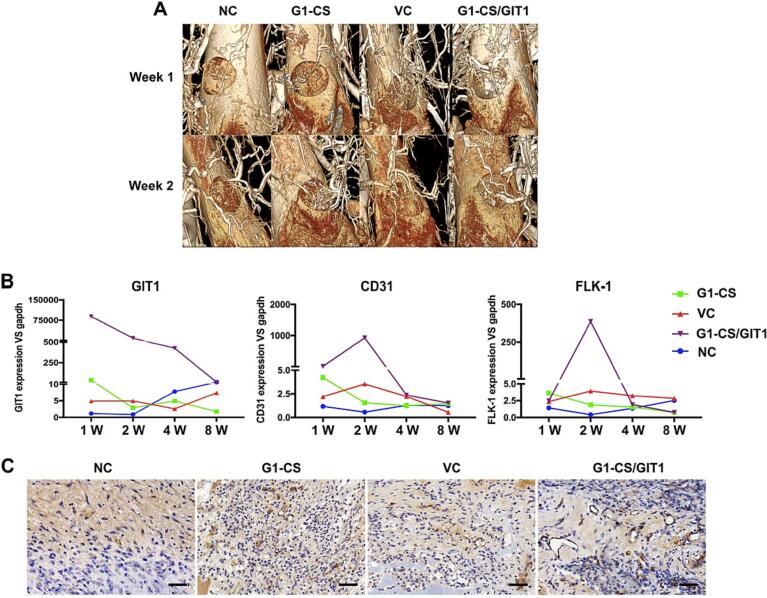
A) Micro-CT analysis of blood vessels around the defect after chromate-based contrast agent perfusion. B) The mRNA expression of angiogenesis-related genes in each group at different time points. C) CD31 immunohistochemical staining of the bone defect at week 2. A brown circle was regarded as a new blood vessel. The scale bar represents 50 μm.

Previous studies have shown that GIT1 plays an important role in the development of pulmonary blood vessels (Pang et al., 2009). GIT1 regulates angiogenic factor secretion in bone marrow MSCs (Li et al., 2019) and regulates stem cell differentiation to promote new blood vessel formation (Majumder et al., 2016). To examine the vascular networks around the bone defect, we used micro-CT analysis combined with a lead chromate-based contrast agent at days 7 and 14 postoperation. As shown in Fig. 6A, at week 1, the **G1-CS**/GIT1 group showed significant sinus formation at the bottom of the defect, while the other three groups showed no significant sinus formation. At week 2, obvious new blood vessels were observed in the bone defects in the **G1-CS**/GIT1 group, while few blood vessels were observed in the **G1-CS** group or the VC group, and no new blood vessels were observed in the NC group.

At 1, 2, 4 and 8 weeks after successful modeling, three SD rats were sacrificed in each group. Total RNA was extracted from the defect site, and the mRNA expression of angiogenesis markers (GIT1, FLK-1 and CD31) was analyzed by RT-PCR. As shown in Fig. 6B, GIT1 expression was significantly enhanced in the **G1-CS**/GIT1 group at week 1, and then gradually decreased. Meanwhile the expression level of GIT1 in the other three groups did not increase significantly, suggesting that DNA transfection was effective in the **G1-CS**/GIT1 group. The mRNA expression of FLK-1 and CD31 in the **G1-CS**/GIT1 group was highly enhanced at week 2, increasing by >300-fold and 1000-fold, respectively. The FLK-1 and CD31 expression levels in the **G1-CS** and VC groups were slightly higher than those in the NC group, but significantly lower than those in the **G1-CS**/GIT1 group. According to the RT-PCR results, we also analyzed vessel numbers using an antibody against CD31 (PECAM1), an endothelial cell marker, to further evaluate angiogenesis of samples at week 2. As shown in Fig. 6C, the brown color showed positive immunohistochemical staining for CD31, and one circle of positive staining represented one new vessel. At week 2, the NC group showed no obvious positive staining. Both the **G1-CS** and VC groups showed a small number of positively-stained circles, while the **G1-CS**/GIT1 group showed a lot of positively-stained circles. These results indicated that few new vessels were formed in the **G1-CS** and VC groups, while a mass of new vessels formed in the **G1-CS**/GIT1 group.

The new blood vessels formed within bone defects can transport nutrients and remove metabolic waste, which is of great significance for the formation of new bone (Xu et al., 2019; Liu et al., 2017; Tabbaa et al., 2014). At week 1 and week 2 postoperatively, we conducted micro-CT scanning and three-dimensional reconstruction after vascular perfusion. We found that at week 1, the **G1-CS**/GIT1 group formed significant blood sinus at the bottom of the bone defect, while the other three groups showed no blood sinus formation. At week 2, significant new blood vessels were detected in the bone defects of the **G1-CS**/GIT1 group, but fewer were evident in the VC and **G1-CS** groups, and no new blood vessels were observed in the NC group. CD31 (Pecam-1) is the integrin protein on the surface of endothelial cells and a specific marker of newly-formed endothelial cells (Lertkiatmongkol et al., 2016). We performed CD31 immunohistochemical staining on samples at 2 weeks postsurgery, and the results further confirmed that there was a large amount of neovascularization in the **G1-CS**/GIT1 group. By using RT-PCR, we analyzed the mRNA levels of the osteogenesis marker genes Runx2, OCN, and BSP and the angiogenesis marker genes CD31 and FLK-1. We found that in the **G1-CS**/GIT1 group, the mRNA levels of CD31 and FLK-1 reached a peak at week 2, which was coincident with that of Runx2, OCN and BSP. Therefore, we considered that GIT1 increased the number of new blood vessels around the bone defect as well as accelerating bone defect healing. Few new blood vessels were detected in the VC group or the **G1-CS** group. This may be due to the network structure of the **G1-CS** hydrogel, which is conducive to vascular growth (Fu et al., 2019; Dos Santos et al., 2019).

### 2.4 MSC differentiation into endothelial cells (ECs) induced by GIT1

**Fig. 7:**
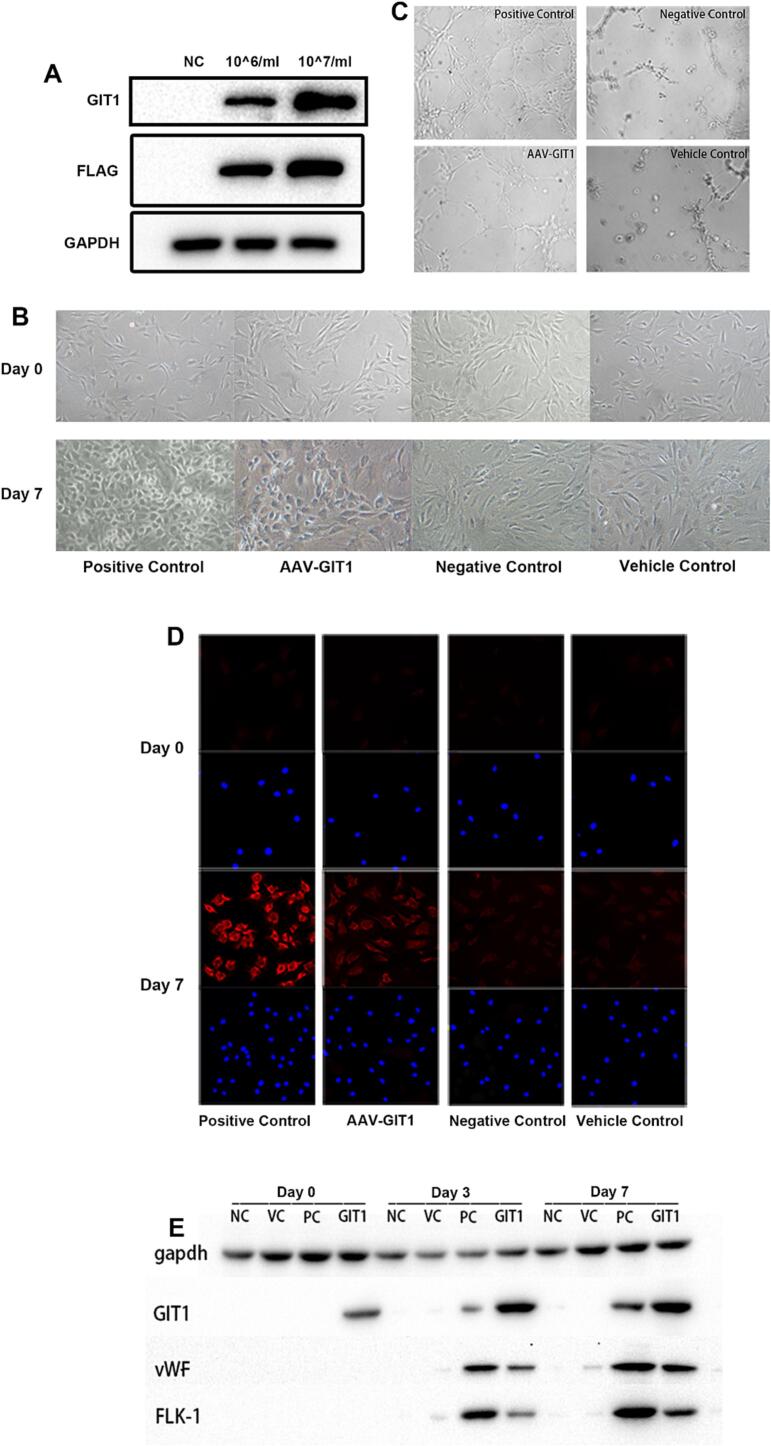
Differentiation of MSCs into endothelial cells by day 7. A) Western blot analysis showed that GIT1 protein expression increased after transfection of AAV-GIT1. B) Morphological changes of differentiating cells. MSCs transfected with AAV-GIT1 presented a cobblestone-like appearance at day 7, as did the positive control group. C) Capillary tube formation assay of differentiating cells. Tube formation was significantly increased in the AAV-GIT1 group and the positive control group. D) Immunofluorescence staining of vWF in differentiating cells. MSCs of the AAV-GIT1 group showed an up-regulation as did the positive control. E) Western blot analysis indicated that protein expression of GIT1, vWF and FLK-1 in the AAV-GIT1 group increased with culture time. All images of cells were taken at 100× magnification.

Due to the low transfection rate of plasmids into MSCs *in vitro*, we constructed an adeno-associated virus (AAV) loaded with GIT1 DNA (AAV-GIT1) and its NC (AAV-NC) to study the effect of GIT1 on MSC differentiation. MSCs were transfected with AAV-GIT1 at different concentrations, and total protein was extracted from cells at 48 h post-transfection and western blotting was used to analyze protein expression. As shown in Fig. 7A, GIT1 expression of transfected MSCs was much higher than that of negative control groups. A viral titer of 10^7^/mL was chosen for use in the following experiments. Then MSCs were cultured for 7 days to observe any changes in cell morphology after transfection with AAV-GIT1. Vascular endothelial growth factor (VEGF) and basic fibroblast growth factor (bFGF) together were used as the positive control, and AAV-NC was used as the vehicle control. As shown in Fig. 7B, MSCs in the positive control group and the AAV-GIT1 group presented a cobblestone-like appearance after 7 days of culture, while those in the VC and NC groups still retained their fusiform shape. We also performed capillary tubelike formation assay to test whether the differentiated MSCs possessed endothelial cell function. As shown in Fig. 7C, capillary tube formation of differentiating MSCs significantly increased in the AAV-GIT1 group compared to that of the VC and NC groups. To further confirm the effect of GIT1 on endothelial differentiation of MSCs, we examined the protein expression of endothelial markers after 7 days of culture. We observed an upregulation of von Willebrand factor (vWF) protein expression in the AAV-GIT1 group compared with the VC and NC groups when analyzed by immunofluorescence staining (Fig. 7D). Furthermore, western blot analysis demonstrated a significant upregulation in protein expression of endothelial-specific markers, including GIT1, vWF and FLK-1 in the AAV-GIT1 group with culture time (Fig. 7E). These results suggested that GIT1 induced the differentiation of MSCs into endothelial cells.

VEGF is a specific marker of vascular endothelial cells, which can induce differentiation of MSCs into vascular endothelial cells (Serra et al., 2019; Almalki and Agrawal, 2017; Ikhapoh et al., 2015). GIT1 is also a specific marker of vascular endothelial cells, and can exert a significant effect on the development of blood vessels (Li et al., 2019; Wang et al., 2009). Therefore, we hypothesized that GIT1 could induce MSCs to differentiate into endothelial cells. The bone defect was connected with the medullary cavity, which contained a large number of mesenchymal stem cells. Thus high expression of GIT1 could induce local MSCs to differentiate into vascular endothelial cells, thereby promoting an increase in the formation of new blood vessels at the bone defect and accelerating bone defect healing.

### 2.5 Differentiation of MSCs into ECs is regulated *via* the Notch signaling pathway

**Fig. 8:**
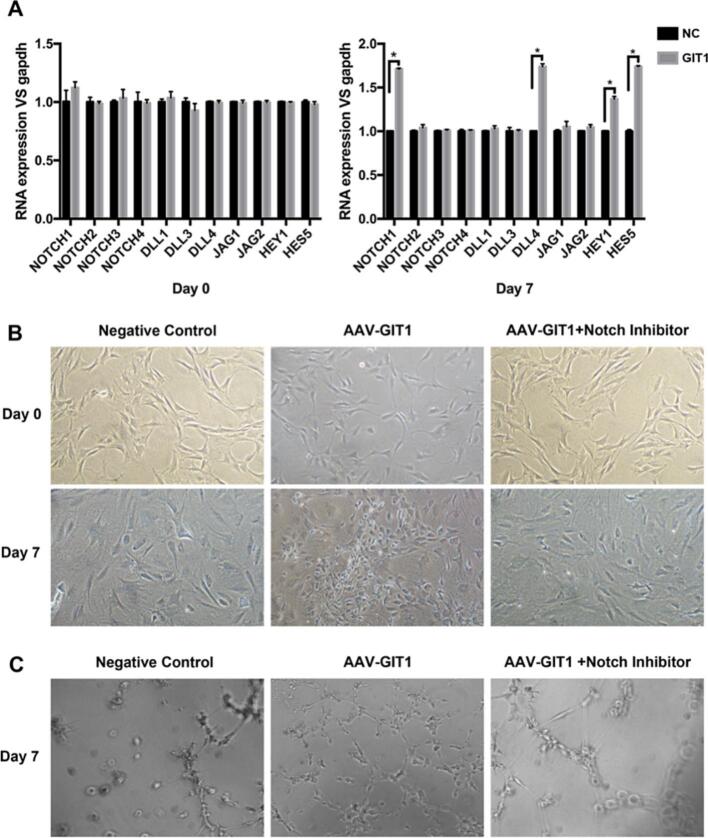
A) Gene expression of Notch signaling components in GIT1-induced MSCs differentiating into ECs. ^⁎^*p* < 0.05. B) Morphological changes of differentiating cells. MSCs treated with AAV and Notch signaling inhibitor retained their fusiform shape. C) Capillary tube formation of cells decreased significantly in the Notch inhibitor-treated group compared to the GIT1 group after 7 days of culture.

To examine whether Notch-DLL4 signaling played a role in the GIT1-induced differentiation of MSCs into endothelial cells, we tested the gene expression of Notch signaling components at day 0 and day 7 by RT-PCR. As shown in Fig. 8A, at day 7, the gene expression of Notch1 and DLL4 increased significantly compared to the control group, while the mRNA levels of Notch 2, 3, 4, DLL1, 3 and Jagged 1, 2 showed no significant difference between the two groups. Hey1 and Hes5 are the downstream targets of Notch signaling which play important roles in cell differentiation (Kaylan et al., 2018; Liu et al., 2010). We also found that the gene expression of Hey1 and Hes5 increased significantly compared to the control group at day 7. These results indicated that Notch signaling was activated during the process of GIT1-induced differentiation of MSCs into endothelial cells. To further confirm this result, we tested the effect of γ-secretase on the GIT1-induced differentiation of MSCs into ECs, which can specifically block the cleavage of Notch and the release of the Notch intracellular domain (NICD). As shown in Fig. 8B, after 7 days of culture, MSCs of the AAV-GIT1 group presented a cobblestone-like appearance, while MSCs of the Notch inhibitor group still retained their fusiform shape. After 7 days of culture (Fig. 8C), capillary tube formation of the Notch inhibitor group decreased significantly compared to that of the AAV-GIT1 group. GIT1 controls stalk cell fate by inhibiting Delta like 4-Notch1 signaling (Majumder et al., 2016). Notch signaling is an evolutionarily-conserved cell fate determinant, which regulates stem cell behavior (Liu et al., 2010). Simvastatin enhances bone marrow stromal cell differentiation into endothelial cells *via* the Notch signaling pathway (Xu et al., 2009). In our study, we found that in the procedure of GIT1-induced MSC differentiation into ECs, the mRNA levels of Notch1, DLL4, Hey1 and Hes5 all increased with culture time, suggesting the activation of Notch signaling. When γ-secretase, a specific blocker of the cleavage of Notch and the production of NICD, was added into the culture medium of MSCs, the differentiation course was significantly blocked. These results further confirmed that the Notch signaling pathway was essential for GIT1-induced MSC differentiation into ECs.

## 3 Conclusion

In summary, an injectable hydrogel with thermoresponsive properties from dendronized chitosan encapsulated with G1T1 (**G1-CS**/GIT1) was investigated as a bone repair material. The results proved that **G1-CS**/GIT1 significantly improved local GIT1 expression by minimally-invasive surgery. High expression of GIT1 promoted osteogenesis and accelerated bone defect repair. We also found that GIT1 induced MSCs to differentiate into ECs, increasing the local vasculature, which also contributed to bone defect repair. This procedure involved activation of the Notch signaling pathway. Overall, we have provided a new strategy for clinical treatment of bone defects.

## 4 Experimental methods

### 4.1 Materials

**G1-CS** hydrogel (prepared as described in a previous report (Zhang et al., 2019a)), MSC medium (ScienCell Research Laboratories, Carlsbad, CA, USA), fetal bovine serum (Gibco Life Technologies, Carlsbad, CA, USA), penicillin-streptomycin (Gibco, 15140122), TRIzol (Invitrogen, Carlsbad, CA, USA), SYBR Green Supermix (Bio-Rad, Hercules, CA, USA), cDNA synthesis kit (Bio-Rad), 4 % paraformaldehyde (Servicebio, Wuhan, China), CD31 antibody (Abcam, Cambridge, UK), vWF antibody (Abcam), CD34 antibody (Abcam), CD90 antibody (Abcam), FLK-1 antibody (Abcam), GIT1 antibody (Abcam), and GAPDH antibody (Abcam). Secondary antibody (Abcam), FITC-conjugated secondary antibody (Jackson ImmunoResearch, West Grove, PA), DAPI (Beyotime Institute of Biotechnology, Jiangsu, China), Alkaline Phosphatase staining kit (Servicebio), PBS (Gibco), Opti-MEM medium (Gibco), AAV-GIT1 and Plasmids-GIT1 [constructed by Obio Technology (Shanghai) Company, Shanghai, China], BD Matrigel (BD Bioscience, San Jose, CA, USA), and Microfil MV-122 (Flow Tech Inc., Carver, MA, USA).

### 4.2 Instruments

Nikon microscope (Type 104; Nikon, Tokyo, Japan), 7900HT Fast Real-Time PCR System (Applied Biosystems, Foster City, CA, USA), Micro-CT (Siemens AG, Erlangen, Germany).

### 4.3 Bone defect rat model

All surgical operations were reviewed and approved by the Institutional Animal Care and Use Committee of Nanjing Medical University (Approval No. IACUC-1705039). Male Sprague-Dawley rats at the age of 8 weeks were used for this study. The surgery was performed as described before (Li et al., 2009). Briefly, the rats were anesthetized with Pentobarbital Sodium, and the surgical field was sterilized with povidone iodine solution. An incision was made at the lateral side of the thigh, the muscles were separated to reach the bone, then a unicortical bone defect (3 mm diameter, 5 mm deep) was created with a drill in the distal region of the femur and the periosteum around the defect was removed, as shown in Fig. 3A. The rats were divided into four groups and the bone defect was treated with **G1-CS** hydrogel, **G1-CS** hydrogel plus empty plasmid, **G1-CS** hydrogel plus plasmid-GIT1, or with nothing as the negative control group. Finally, the muscle and the skin were sutured with silk threads.

### 4.4 Angiography of bone defect region

At days 7 and 14 postsurgery, the vascular networks around the defect field were examined using micro-CT analysis combined with perfusion of a lead chromate-based contrast agent as described previously (Yin et al., 2014). In brief, the rats were sacrificed, the vasculature was flushed through *via* the abdominal aorta with heparinized saline and 4 % paraformaldehyde, and then perfused with Microfil MV-122 (Flow Tech) contrast medium, a radiopaque silicone rubber compound containing lead chromate. The rats were stored at 4 °C overnight and then examined by micro-CT (Siemens) the next day at a resolution of 9.5 μm.

### 4.5 Histological tissue analyses

After micro-CT analysis, the femurs were decalcified with 10 % EDTA, and subsequently embedded in paraffin. Coronal sections 5-μm thick were cut, the slices were deparaffinized, rehydrated and then stained with H&E, for ALP and total collagen.

### 4.6 Immunohistochemistry

The sections were deparaffinized and heated in citrate buffer (2.1 M tri‑sodium citrate, PH 6.0) at 100 °C for 30 min to retrieve antigen, then incubated with blocking buffer containing 2 % goat serum and 3 % bovine serum albumin for 2 h. After that, the slides were incubated at 4 °C overnight with polyclonal primary antibodies to CD31 (1:100). After washing in PBS three times, the sections were incubated with a secondary antibody (1:200) for 1 h at room temperature. Images were captured under a Nikon microscope.

### 4.7 RNA extraction and real-time PCR

At 1, 2, 4 and 8 weeks postsurgery, the rats were sacrificed and the femurs were removed. The defect zones were collected and ground into powder in liquid nitrogen. Then 1 mL TRIzol (Invitrogen) was added to the powder, and the mixture were stored at −80 °C for 24–48 h to permit the complete dissociation of nucleoprotein complexes. After that, the mixture was thawed at room temperature and experiments were carried out following the instructions to extract total RNA. RT-PCR was performed on an Applied Biosystems 7900 Real-Time PCR system using SYBR green master mix and primers are listed in **Table S1**. The comparative C_T_ method was used to determine the relative expression levels of the genes of interest. In detail, the target gene expression was first normalized to that of the housekeeping gene encoding GAPDH, and then normalized by gene expression measured in the negative control group at 1 week.

### 4.8 Primary cell culture and identification

Primary MSCs were isolated from the bone marrow of newborn rats (day 7). The rats were sacrificed, the femurs and tibias were harvested, the bone marrow was flushed out and bone marrow cells were cultured in MSC medium at 37 °C in 5 % CO_2_. After 24 h, the medium was refreshed to remove nonadherent cells, and the adherent cells were cultured and expanded for three passages. At passage three, MSCs were washed with PBS three times and digested with trypsin (without EDTA), to generate a single-cell suspension with cell concentration of 10^6–10^7/mL. After centrifugation at 1500 rpm for 3 min, the supernatant was discarded, and then the cells were resuspended in PBS. After another centrifugation at 1500 rpm for 3 min, the supernatant was discarded again. CD34, CD90 and their isotype control antibodies were added into each tube and incubated for 30 min at room temperature in the dark. Flow cytometric analysis was used to detect the positive expression of the markers. The results are shown in **Fig. S3**.

### 4.9 AAV transfection

MSCs at passage three were collected and resuspended in 24-well plates (1 × 10^5^ cells/well). When the cell density reached 50–70 %, the medium was replaced with 250 μL Opti-MEM medium containing AAV. Then the cells were incubated at 37 °C in 5 % CO_2_ for 2 h followed by addition of 250 μL growth medium to each well. After 24 h of incubation, the medium was replaced with fresh growth medium.

### 4.10 Plasmid transfection

The 293 T cells or MSCs were cultured in 24-well plates. When the cell density reached 50–70 %, the medium was replaced with 250 μL Opti-MEM medium containing plasmids. Then the cells were incubated at 37 °C in 5 % CO_2_ for 4 h after which the medium was replaced with growth medium.

### 4.11 Cell immunofluorescence

Cells with 50–70 % fusion were washed with PBS three times, fixed in 4 % paraformaldehyde for 10 min, diluted with 0.3 % Triton X-100 in PBS for 10 min, and then blocked with PBS-diluted goat serum for 1 h. The cells were incubated with anti-mouse vWF antibody at 4 °C overnight and then treated with goat anti-mouse fluorescent secondary antibody for 1 h. After that, cells were washed with PBS three times followed by DAPI nuclear staining. The immunofluorescent density of cells was calculated under a light microscope.

### 4.12 Western blotting

Cell lysates were fractionated by sodium dodecyl sulfate polyacrylamide gel electrophoresis (SDS-PAGE) and transferred to polyvinylidene difluoride (PVDF) membranes. The blocked membranes were incubated with appropriate antibodies, and the immunoreactive bands were analyzed with a chemiluminescent reagent.

### 4.13 Tube formation assay

The tube formation assay was performed according to the instructions of BD Matrigel. Briefly, 50 μL Matrigel was added to each well of a 96-well plate and stored at 37 °C for 30 min to polymerize. After trypsinization, the collected cells were resuspended and plated onto the layer of Matrigel (2 × 10^4 cells/well). Then the Matrigel cultures were incubated at 37 °C for 6 h before photographing under a Zeiss microscope.

### 4.14 Statistical analyses

Mean values and standard deviation were calculated for the relative gene expression. Differences between different groups were determined by One-way ANOVA test. *P* values < 0.05 were considered significantly different. Statistical analysis was carried out by using SPSS 11.0 software (SPSS Inc., Chicago, IL, USA).

## CRediT authorship contribution statement

**Lin Cheng:** Data curation, Formal analysis, Funding acquisition, Investigation, Writing – original draft, Writing – review & editing. **Zhimin Zhou:** Data curation, Formal analysis, Investigation. **Qingqing Li:** Data curation, Software, Writing – original draft. **Wen Li:** Formal analysis, Supervision, Writing – review & editing. **Xin Li:** Data curation. **Gen Li:** Software. **Jin Fan:** Conceptualization, Funding acquisition, Supervision, Project administration. **Lipeng Yu:** Supervision, Validation, Writing – review & editing. **Guoyong Yin:** Funding acquisition, Project administration, Supervision, Writing – review & editing.

## Declaration of competing interest

There are no conflicts to declare.

## Data Availability

All data included in this study are available upon request by contact with the corresponding author.

## Appendix A Supplementary data

Supplementary material Image 1
