# Supplementary material for: Dendronized chitosan hydrogel with GIT1 to accelerate bone defect repair through increasing local neovascular amount
Source: Bone Rep. 2023 Aug 30;19:101712. doi: 10.1016/j.bonr.2023.101712 (PMC10511783; doi:10.1016/j.bonr.2023.101712)
Supplement: Supplementary file 1 — Supplementary material [file mmc1.docx]

Supplementary Information

Dendronized Chitosan Hydrogel with GIT1 to Accelerate Bone Defect Repair through Increasing Local Neovascular Amount

Lin Cheng,^#^ Zhimin Zhou,^#^ Yihao Wu,^#^ Qingqing Li, Xin Li, Wen Li, Jin Fan,^*^ Lipeng Yu,^*^ and Guoyong Yin^*^

**Table S1**. Primer sequences

| Gene | Forward primer (5'—3') | Reverse primer (5'—3') |
| --- | --- | --- |
| Rat-GAPDH | ACAGCAACAGGGTGGTGGAC | TTTGAGGGTGCAGCGAACTT |
| Rat-Runx2 | CGCCTCACAAACAACCACAG | AATGACTCGGTTGGTCTCGG |
| Rat-BSP | AGGCTACGAGGGTCAGGATT | CCGTAGCACCATTCCACACT |
| Rat-OCN | ATTGTGACGAGCTAGCGGAC | TCGAGTCCTGGAGAGTAGCC |
| Rat-GIT1 | CCGCACACCCATTGACTATG | CCGAGATCTGTCAGCCATCT |
| Rat-CD31 | GGTGTACAACGTCTCCTCCA | CCACCTTCTGTCACCTCCTT |
| Rat-FLK-1 | AGGGCCTCTCATGGTGATTG | TCCTCCACAAAACCTGAGCT |
| Rat-Notch1 | CAAGACCTTGAACCTCGGGTA | TGGGTAGAACACTCCACACACA |
| Rat-Notch2 | TGTTACCTACCACAACGGCAC | TGACAGCGGTTCTTCTCACAA |
| Rat-Notch3 | ATCTCGCTCTCATCCCTTTATCT | TGACCAACTCTGTCACACTTCAA |
| Rat-Notch4 | GAAGCGGATGAATGTCGGAGT | GATGGGGCTACACAAGGGAAC |
| Rat-DLL1 | GAAGAAGAGGAAACCGAAAGTTGT | CGCCCTTGACTCTCCTAGAGTATC |
| Rat-DLL3 | TGGTGTCTTCGAGCTACAAATTCA | TCAGGCAGACCCTGAAGAAGAG |
| Rat-DLL4 | GCAGAACCACACACTGGACTATAA | GAAGTGGCACCTTCTCTCCTAA |
| Rat-Jagged1 | GCCGCCATAGGTAGAGTTTGA | TGGAAGCCAGCTTGTCGAA |
| Rat-Jagged2 | TGCCATCACCCAGAGAGGAA | CAGCACACTGAACACGCTACAC |
| Rat-Hey1 | GTTATCTGAGCATCATTGAAGGACT | CCAAGGAATGTGTCCGAGG |
| Rat-Hes5 | CAGCATTGAGCAGCTGAAACT | TCCAGGATGTCGGCCTTCT |


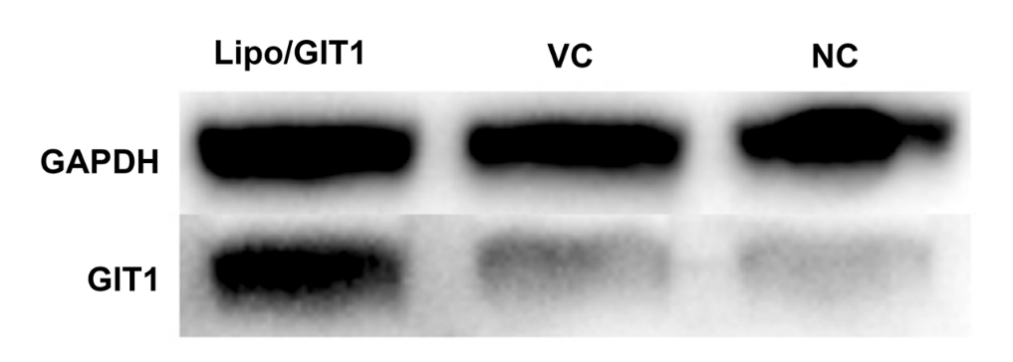


**Figure S1**. GIT1 protein expression of 293T cells after transfection with plasmid-GIT1.


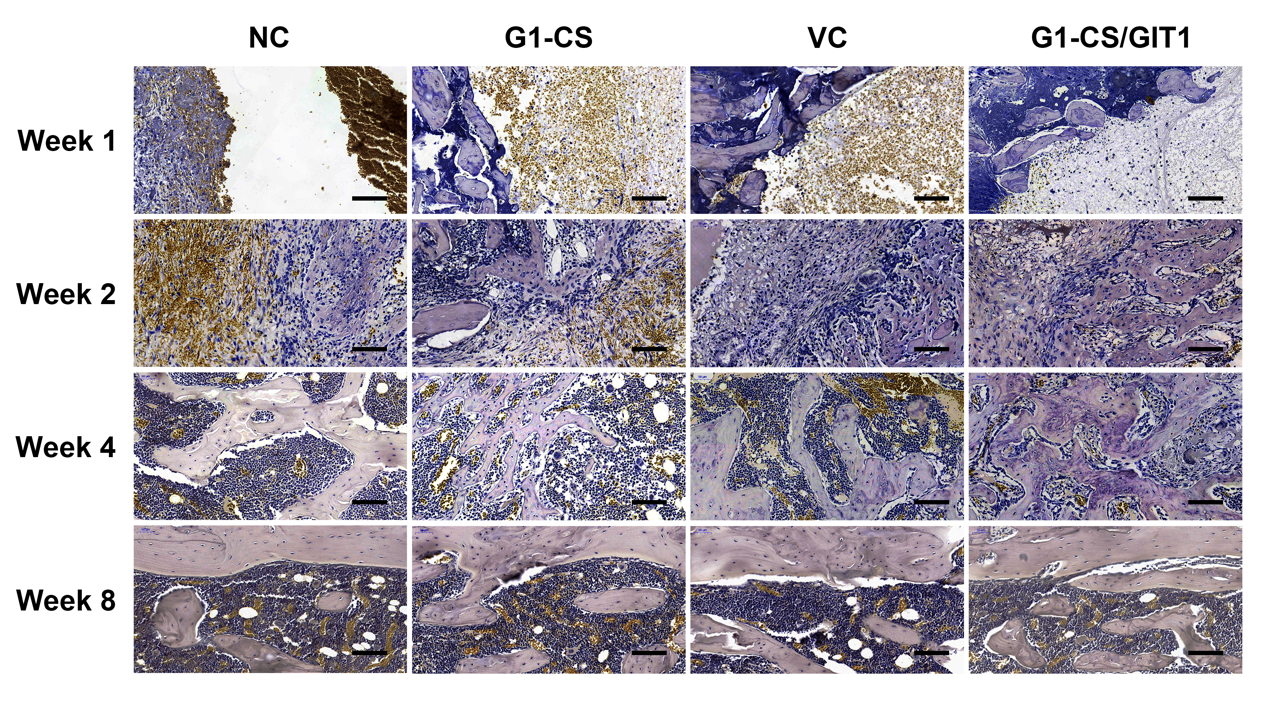


**Figure** **S2**. ALP staining of the defect area of different groups at different time points. The scale bar represents 100 µm.


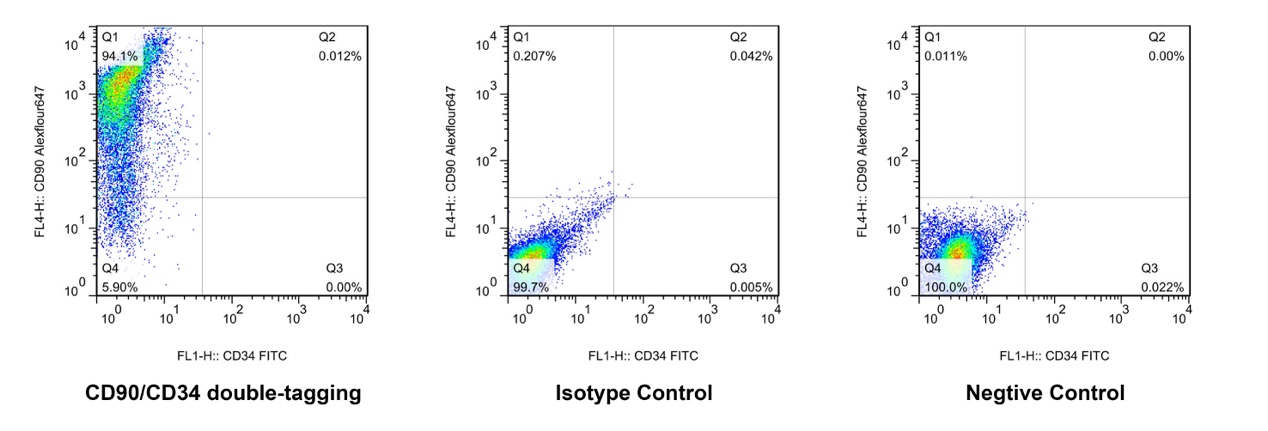


**Figure S3**. Flow cytometric analysis of the positive marker CD90 and negative marker CD34 expressed by MSCs. The positive expression rates of CD90 and CD34 were 94.1% and 0.00%, respectively.
